# Prevalence of and Risk Factors Associated with Polymerase Chain Reaction-Determined *Plasmodium falciparum* Positivity on Day 3 after Initiation of Artemether–Lumefantrine Treatment for Uncomplicated Malaria in Bagamoyo District, Tanzania

**DOI:** 10.4269/ajtmh.18-0729

**Published:** 2019-03-11

**Authors:** Richard Mwaiswelo, Billy Ngasala, Irina Jovel, Weiping Xu, Erik Larsson, Maja Malmberg, Jose Pedro Gil, Zul Premji, Bruno P. Mmbando, Andreas Mårtensson

**Affiliations:** 1 Department of Parasitology and Medical Entomology, Muhimbili University of Health and Allied Sciences, Dar es Salaam, Tanzania;; 2 Department of Microbiology and Parasitology, Hubert Kairuki Memorial University, Dar es Salaam, Tanzania;; 3 Department of Women’s and Children’s Health, International Maternal and Child Health (IMCH), Uppsala Universitet, Uppsala, Sweden;; 4 Department of Microbiology, Tumor and Cell Biology, Karolinska Institutet, Stockholm, Sweden;; 5 Section of Virology, Department of Biomedical Sciences and Veterinary Public Health, Swedish University of Agricultural Sciences, Uppsala, Sweden;; 6 SLU Global Bioinformatics Centre, Department of Animal Breeding and Genetics, Swedish University of Agricultural Sciences, Uppsala, Sweden;; 7 Drug Resistance Unit, Division of Pharmacogenetics, Department of Physiology and Pharmacology, Karolinska Institutet, Stockholm, Sweden;; 8 Aga Khan University Hospital, Nairobi, Kenya;; 9 Tanga Centre, National Institute for Medical Research, Tanga, Tanzania

## Abstract

Prevalence of and risk factors associated with polymerase chain reaction (PCR)-determined *Plasmodium falciparum* positivity were assessed on day 3 after initiation of treatment, pre-implementation and up to 8 years post-deployment of artemether–lumefantrine as first-line treatment for uncomplicated malaria in Bagamoyo district, Tanzania. Samples originated from previously reported trials conducted between 2006 and 2014. Cytochrome b-nested PCR was used to detect malaria parasites from blood samples collected on a filter paper on day 3. Chi-square and McNemar chi-squared tests, logistic regression models, and analysis of variance were used as appropriate. Primary outcome was based on the proportion of patients with day 3 PCR-determined *P. falciparum* positivity. Overall, 256/584 (43.8%) of screened patients had day 3 PCR-determined positivity, whereas only 2/584 (0.3%) had microscopy-determined asexual parasitemia. Day 3 PCR-determined positivity increased from 28.0% (14/50) in 2006 to 74.2% (132/178) in 2007–2008 and declined, thereafter, to 36.0% (50/139) in 2012–2013 and 27.6% (60/217) in 2014. When data were pooled, pretreatment microscopy-determined asexual parasitemia ≥ 100,000/µL, hemoglobin < 10 g/dL, age < 5 years, temperature ≥ 37.5°C, and year of study 2007–2008 and 2012–2013 were significantly associated with PCR-determined positivity on day 3. Significant increases in *P. falciparum* multidrug resistance gene 1 N86 and *P. falciparum* chloroquine resistant transporter K76 across years were not associated with PCR-determined positivity on day 3. No statistically significant association was observed between day 3 PCR-determined positivity and PCR-adjusted recrudescence. Day 3 PCR-determined *P. falciparum* positivity remained common in patients treated before and after implementation of artemether–lumefantrine in Bagamoyo district, Tanzania. However, its presence was associated with pretreatment characteristics. Trials registration numbers: NCT00336375, ISRCTN69189899, NCT01998295, and NCT02090036.

## INTRODUCTION

Artemisinin-based combination therapy (ACT) is the mainstay treatment for uncomplicated *Plasmodium falciparum* malaria globally.^1^ Artemisinin-based combination therapy consists of fast-acting artemisinin derivatives and a long-acting partner drug with a different mode of action. Initially, artemisinin rapidly clears a large number of asexual parasites before the long-acting partner drug eliminates the residual parasitemia.^2^ For artemisinin-sensitive *P. falciparum* parasites, microscopy-determined parasite clearance usually occurs within 48 hours in most of the patients treated with ACT.^3^ Artemisinin-resistant *P. falciparum* parasites, on the other hand, are phenotypically characterized by a slow clearance rate.^4^ Microscopy-based *P. falciparum* positivity rate on day 3 after initiation of ACT treatment is considered an important determinant of delayed parasite clearance, and if the positivity rate exceeds 10%, this is considered an alert for artemisinin resistance.^5^ The resistance has been linked to polymorphisms in the Kelch gene located on chromosome 13 (*k13*) of *P. falciparum* parasites.^6^

Artemisinin resistance has been reported from four countries in the Mekong region, Southeast Asia.^4,7,8^ In Africa, despite recent case reports of suspected resistance,^9,10^ *Pfk13* polymorphisms are rare and do not include those previously described and associated with delayed *P. falciparum* clearance in Southeast Asia.^11,12^ Artemisinin-based combination therapy has also remained highly efficacious in Africa, with rapid microscopy-determined clearance of the asexual parasites within 48 hours after initiation of treatment.^13–15^ However, few African studies have assessed the presence of residual polymerase chain reaction (PCR)-determined *P. falciparum* positivity on day 3 after initiation of ACT treatment,^16,17^ a phenomenon that may indicate reduced susceptibility of *P. falciparum* against artemisinin derivatives and thereby a potential early warning marker of artemisinin resistance.^16^

Nonetheless, several pretreatment host and parasite characteristics including initial parasite density, age of the patient, and hemoglobin level influence parasite clearance after treatment with ACT.^18–20^ Higher initial microscopy-determined asexual parasitemia (≥ 100,000 parasites/µL of blood) and anemia, that is hemoglobin < 10 g/dL, are associated with slow parasite clearance.^19,21^ Conversely, in high malaria-endemic areas, malaria immunity increases with age, whereby children aged < 5 years have low acquired immunity against *P. falciparum* infection and are, therefore, prone to slower parasite clearance than older individuals.^18^ However, it is not well understood whether these factors are responsible for the previously observed day 3 PCR-determined *P. falciparum* positivity in Africa, rather than reduced parasite susceptibility against artemisinins.^16,17^

Therefore, the aim of this study was to evaluate the prevalence of, and risk factors associated with, PCR-determined *P. falciparum* positivity on day 3 after artemether–lumefantrine treatment initiation in clinical trials conducted between 2006 and 2014, covering pre-implementation and 8 years post-implementation of artemether–lumefantrine as first-line treatment for uncomplicated *P. falciparum* malaria in Bagamoyo district, Tanzania.

## MATERIAL AND METHODS

### Study area and design.

The blood samples used for PCR analyses in this report originated from four clinical trials conducted in Fukayosi and Yombo dispensaries in Bagamoyo district, Tanzania, between 2006 and 2014.^22–24^ The district has a tropical climatic condition, with annual rainfall averaging 1,100 mm. Malaria transmission is high and stable throughout the year, with a slight variation between rainfall and dry season.^25^ *Plasmodium falciparum* is the predominant malaria species and *Anopheles gambiae* sensu stricto is the main vector.^26^ Artemether–lumefantrine was adopted in Tanzania as a first-line treatment for uncomplicated *P. falciparum* malaria in November 2006. The use of long-lasting insecticide-treated mosquito nets is the major vector control method in the study area.^27^

This was a retrospective study designed to assess the prevalence of, and risk factors associated with, PCR-determined *P. falciparum* positivity on day 3 after initiation of artemether–lumefantrine treatment among patients with acute uncomplicated *P. falciparum* malaria before and after the implementation of artemether–lumefantrine treatment policy. Four clinical trials were included in the analysis: 1) an artemether–lumefantrine pharmacokinetics and pharmacodynamics trial, 2006,^22^ 2) the supervised arm of an artemether–lumefantrine efficacy (supervised) versus effectiveness (unsupervised) trial, 2007–2008,^23^ 3) an artemether–lumefantrine efficacy trial, 2012–2013,^15^ and 4) both arms of an artemether–lumefantrine versus artemether–lumefantrine plus 0.25 mg/kg single dose of primaquine efficacy and safety trial, 2014.^24^ The primaquine arm was included in the analysis because no statistically significant differences in microscopy-determined parasite clearance, prevalence of PCR-determined *P. falciparum* positivity on day 3, prevalence of *P. falciparum* multidrug resistance gene 1 (*Pfmdr1*) N86 and *P. falciparum* chloroquine resistant transporter (*Pfcrt*) K76, and crude and PCR-adjusted cure rates were observed between the artemether–lumefantrine and artemether–lumefantrine plus primaquine arms.^28^

The 2006 trial was conducted in June, few months before deployment of artemether–lumefantrine in Bagamoyo district, whereas the 2007–2008, 2012–2013, and 2014 trials were conducted 1–2, 6–7, and 8 years, respectively, after wide scale use of artemether–lumefantrine as treatment policy in the study area.

### Study population, treatment, and procedures.

The study subjects were recruited from patients with symptoms and signs compatible with acute uncomplicated *P. falciparum* malaria attending the dispensaries as previously described.^22–24^ Patients with microscopically confirmed *P. falciparum* mono-infection and who fulfilled all the inclusion criteria and none of the exclusion criteria were enrolled and treated under supervision with standard weight-based six-dose artemether–lumefantrine regimen according to the national treatment guidelines in Tanzania. For patients allocated with artemether–lumefantrine plus primaquine treatment, an additional single 0.25 mg/kg bodyweight primaquine dose was administered concomitantly with the first artemether–lumefantrine dose.^24^ Patients were observed for 30 minutes after the intake of each drug dose; and treatment was readministered in case of vomiting within this period. Patients with fever greater than 38.5°C were treated with paracetamol.

Follow-up for clinical and laboratory assessments was performed on days 0, 1, 2, and 3, for the 2006 trial; 0, 1, 2, 3, 7, 14, 21, and 28 or any day of recurrent illness, for the 2014 trial; 0, 1, 2, 3, 7, 14, 21, 28, and 42, for the 2012–2013 trial; and 0, 1, 2, 3, 7, 14, 21, 28, 35, 42, 49, and 56 for the 2007–2008 trial. Every clinical assessment included the history of clinical symptoms, possible adverse events, concomitant drug consumption, clinical examination, and measurement of axillary temperature. Patients who missed a scheduled follow-up visit and who could not be found despite efforts to trace them at their homes were considered lost to follow-up and were consequently withdrawn.^29^ Patients with repetitive vomiting of the trial drug were managed according to national guidelines and followed up until recovery.^27^

Laboratory assessment involved collection of a finger-prick blood sample from patients in all four studies for hemoglobin quantification, thick blood films for the microscopical determination of the presence of *P. falciparum*, and parasite density thin films for the species identification for the 2012–2013 and 2014 cohorts, and 100 µL of blood on a filter paper (3MM Whatman, Sigma-Aldrich Chemie Gmbh, Munich, Germany) for molecular genotyping of asexual parasites.

Hemoglobin concentration was measured using a portable spectrophotometer HemoCue Hb 201+ (HemoCue AB, Ängelholm, Sweden), as previously described.^24^ The blood films were stained using 10% giemsa and read by qualified microscopists, as previously described.^22–24^ The filter papers were labeled, air-dried at room temperature for 3–4 hours, packed in an individual plastic bag, and transported to Karolinska Institutet, Sweden, for molecular analysis.

### Molecular analysis.

Genomic DNA was extracted from dried blood spots collected on a filter paper on days 0, 3, and, when applicable, on the day of microscopy-determined recurrent *P. falciparum* infection during follow-up using ABI PRISM^®^ 6100 Nucleic Acid Prep Station (Applied Biosystems, Fresno, CA) for 2006, and on day 0 and the day of recurrent infection for 2007–2008 samples, whereas, a 10% chelex-100 (Bio-Rad Laboratories Ltd., Hertfordshire, United Kingdom) method was used for the day 3 samples for 2007–2008 trial, and days 0, 3, and the day of recurrent infection samples for the 2012–2013 and 2014 trials.^22,30^ The detection limit of the ABI PRISM 6100 extraction method has been estimated to be 200 parasites/μL and that of the chelex-100 method to be 2 parasites/μL.^31^

Genomic DNA extracted from filter papers collected on day 3 after artemether–lumefantrine treatment initiation from all four studies was used to detect PCR-determined *P. falciparum* positivity using cytochrome-b nested PCR, as previously described.^32^ Briefly, 5 μL of the extracted DNA was amplified with 0.1 μL of 100 pmol/μL of each primer, 2.5 μL of 2 mM dNTPs (Sigma Aldrich Chemie Gmbh, Munich, Germany), 2 μL of 25 mM MgCl_2_, and 0.125 μL of 5 U/μL Taq DNA polymerase (Sigma Aldrich) and 10.175 μL of dH_2_O, using the following cycling program: 3 minutes at 95°C, then 40 cycles of 15 seconds at 95°C, 60 seconds at 60°C, 70 seconds at 72°C, and a final extension of 5 minutes at 72°C. For the nested-PCR, 3 μL of primary PCR product was amplified with 0.05 μL of 100 pmol/μL of each primer, 2.5 μL of 2 mM deoxynucleoside triphosphates (dNTPs) 2 μL of 25 mM MgCl_2_, and 0.125 μL of 5 U/μL Taq DNA polymerase and 12.175 μL of dH_2_O, using the following cycling program: 3 minutes at 95°C, then 40 cycles of 15 seconds at 95°C, 60 seconds at 60°C, 60 seconds at 72°C, and a final extension of 5 minutes at 72°C. *Pfmdr1* N86Y and *Pfcrt* K76T were genotyped using nested PCR followed by restriction fragment length polymorphism using *Apo*I (Thermo Fisher Scientific, Waltham, MA) restriction enzyme.^33^

Blood samples from patients with recurrent infection during follow-up (2007–2008, 2012–2013, and 2014 trials) were further genotyped to differentiate recrudescence (treatment failure) from reinfection (new infection) by stepwise genotyping of *P. falciparum* block 3 of merozoite surface proteins (msp) 2, block 2 of msp 1, and region II (RII) of glutamate-rich protein (glurp).^34^

The amplicons were loaded, respectively, on GelRed^™^ (Biotium, Inc., Hayward, CA) stained agarose, separated by electrophoresis, and visualized under Ultra Violet transillumination (Gel Doc^™^ System; Bio-Rad, Hercules, CA) using Image Lab^™^ software (Bio-Rad). For merozoite surface protein (msp) 1 1, 2, and glurp, alleles in each family were considered the same if fragments’ size was within 20 base pair intervals. Patients with microscopy-determined recurrent *P. falciparum* infection, but with negative PCR results, were considered to have uncertain PCR-adjusted outcome and were consequently excluded from the final analysis. Recrudescence was defined as the presence of at least one matching allelic band, and reinfection was defined as the absence of any matching allelic band at baseline and on the day of parasite recurrence.^34^

### Study endpoints.

The primary outcome was the proportion of patients with PCR-determined *P. falciparum* positivity on day 3 after treatment initiation with artemether–lumefantrine between 2006 and 2014. Polymerase chain reaction–determined *P. falciparum* positivity was predefined as parasites that were detectable by cytochrome b-nested PCR. The secondary outcomes were as follows: prevalence of day 3 PCR-determined *P. falciparum* positivity across years between 2006 and 2014, the proportion of *Pfmdr1* N86Y and *Pfcrt* K76T on days 0 and 3, and their association with the presence of day 3 PCR-determined *P. falciparum* positivity across years; change in the proportion of *Pfmdr1* N86Y and *Pfcrt* K76T between days 0 and 3 across years; the association between baseline characteristics including age < 5 years, microscopy-determined pretreatment asexual parasitemia (≥ 100,000/µL of blood), fever (≥ 37.5°C), anemia (hemoglobin < 10 g/dL), *Pfmdr1* N86 and *Pfcrt* K76, and PCR-determined *P. falciparum* positivity on day 3; and the association between PCR-determined *P. falciparum* positivity on day 3 and crude recurrent infection, as well as PCR-adjusted recrudescence during follow-up after artemether–lumefantrine treatment.

### Ethical considerations.

Good Clinical Practice, Declaration of Helsinki, and applicable regulatory requirements in Tanzania were adhered to during the conduct of the trials. Approvals to conduct the respective trials were obtained from the Ethics Committees of the Muhimbili University of Health and Allied Sciences, National Institute for Medical Research, Tanzania Food and Drug Authority, and the Regional Ethics Committee, Stockholm, Sweden. Written informed consent was obtained from all patients and parents/guardians of patients aged < 18 years, before enrollment. The trials are registered with numbers: NCT00336375, ISRCTN69189899, NCT01998295, and NCT02090036.

### Statistical analysis.

Data were double entered and analyzed using R version 3.2.3 (R Foundation, Vienna, Austria). Independent proportions were compared using chi-squared test, whereas paired proportions were compared using McNemar chi-squared test. Age was negatively skewed and was, therefore, transformed using log. Trend of change in proportion of day 3 PCR-determined *P. falciparum* positivity, *Pfmdr1* N86Y, and *Pfcrt* K76T across years was assessed using logistic regression model. Univariate and multivariate logistic regression models were used to assess the association of pretreatment risk factors with both residual parasites and recurrent infection. The association between pretreatment characteristics and day 3 PCR-determined *P. falciparum* positivity was assessed both for each trial individually and when data from all trials were pooled. The 2006 trial had a follow-up of 3 days only, and hence excluded in the analysis to assess the association of day 3 PCR-determined *P. falciparum* positivity and recurrent infection, including both crude microscopy-based recurrent infection and PCR-adjusted recrudescence, during follow-up after artemether–lumefantrine treatment. Mean differences between groups were compared using the analysis of variance. Data were censored at the time of withdrawal in case of patient lost to follow-up, withdrawal of consent, and PCR-adjusted reinfection or uncertain PCR outcome. A statistical significance level was set at *P* ≤ 0.05.

## RESULTS

### Characteristics of the study participants.

A total of 584 patients were screened for PCR-determined *P. falciparum* positivity on day 3, in which, 256 (43.8%) were PCR positive. Of the PCR-positive patients, only two (0.3%) had microscopy-determined asexual parasites on day 3. Pretreatment characteristics of the study participants are presented in Table 1. Participants in 2007–2008 were the youngest, had the lowest mean hemoglobin, and the highest microscopy-determined asexual mean parasite density, whereas in 2014 study, participants had the highest median age and hemoglobin levels, and the lowest mean parasite density. However, when a separate analysis was performed for children aged < 5 years, there was a slight change in the parameters, whereby children in 2006 had slightly lower mean age and hemoglobin concentration, whereas those in 2012–2013 had the highest microscopy-determined asexual parasite density (Table 1).

**Table 1:** Pretreatment characteristics of the study participants

Characteristic	Year of study				Statistical test
	2006	2007–2008	2012–2013	2014	
All ages	*N* = 50	*N* = 172	*N* = 139	*N* = 217	
Age (years), median (IQR)	4.0 (2.0–6.0)	2.7 (1.8–3.8)	4.5 (2.3–6.7)	9.0 (5.0–18.0)	*x*^2^_3_ = 233, *P* < 0.001
Gender (female), *n* (%)	31 (62.0)	92 (51.7)	77 (55.4)	108 (49.8)	*x*^2^ = 3.0, *P* = 0.39
Weight (kg), mean (SD)	14.3 (5.5)	12.2 (3.0)	16.8 (5.9)	32.3 (18.4)	*F* = 110, *P* < 0.001
Temperature (°C), mean (SD)	38.5 (1.3)	38.6 (1.2)	38.2 (1.3)	38.3 (1.2)	*F* = 4.2, *P* = 0.006
Hemoglobin (g/dL), mean (SD)	10.1 (1.7)	9.6 (2.0)	10.5 (1.8)	11.2 (1.5)	*F* = 29, *P* < 0.001
Parasitemia/µL, and geometric mean (95% CI) by microscopy	21,647 (14,371–32,606)	42,276 (36,291–49,249)	23,839 (18,335–30,995)	8,413 (6,215–11,392)	*F* = 28.8, *P* < 0.001
Febrile (≥ 37.5°C), *n* (%)	37 (74.0)	146 (82.0)	88 (63.3)	165 (76.0)	*x*^2^ = 3.0, *P* = 0.091
Anemia (hemoglobin < 10 g/dL), *n* (%)	22 (44.0)	101 (57.4)	12 (8.6)	44 (20.3)	*x*^2^ = 105, *P* < 0.001

Patients aged < 5 years	*N* = 33	*N* = 172	*N* = 76	*N* = 39	
Age (years), median (IQR)	2.4 (1.7–4.0)	2.6 (1.8–3.8)	2.5 (1.4–3.6)	3.0 (2.0–4.0)	*x*^2^_3_ = 1.74, *P* = 627
Gender (female), *n* (%)	18 (54.5)	90 (51.4)	46 (60.5)	17 (43.6)	*X*^2^ = 3.3, *P* = 0.35
Weight (kg), mean (SD)	11.1 (2.0)	12.1 (3.0)	15.3 (6.9)	13.2 (2.5)	*F* = 11.7, *P* < 0.001
Temperature (°C), mean (SD)	38.8 (1.4)	38.6 (1.2)	38.2 (1.3)	38.7 (1.2)	*F* = 3.2, *P* = 0.025
Hemoglobin (g/dL), mean (SD)	9.4 (1.4)	9.6 (2.0)	10.2 (1.9)	10.4 (1.3)	*F* = 3.7, *P* = 0.012
Parasitemia/µL, and geometric mean (95% CI) by microscopy	23,889 (14,451–39,491)	42,276 (36,291–49,249)	49,773 (28,503–86,896)	10,816 (5,076–23,046)	*F* = 10.7, *P* < 0.001
Febrile (≥ 37.5°C), *n* (%)	26 (78.8)	143 (81.7)	46 (60.5)	32 (82.1)	*X*^2^ = 4.4, *P* = 0.035
Anemia (hemoglobin < 10 g/dL), *n* (%)	20 (60.6)	100 (57.8)	11 (14.5)	14 (35.9)	*X*^2^ = 32.9, *P* < 0.001

### Prevalence of PCR-determined *P. falciparum* positivity on day 3 across years

**Figure 1.**
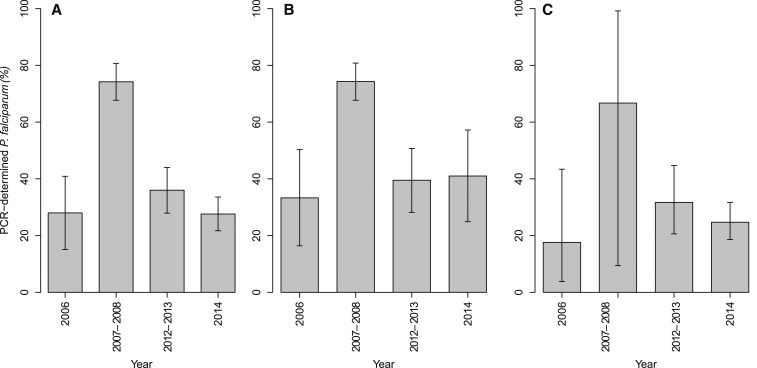
Distribution of patients with PCR-determined parasitemia on day 3 from 2006 to 2014. (**A**) All ages, (**B**) Patients < 5 years, (**C**) Patients > 5 years.

The prevalence of PCR-determined *P. falciparum* positivity on day 3 increased from 28.0% (14/50) in 2006 to 74.2% (132/178) in 2007–2008 and declined, thereafter, to 27.6% (60/217) in 2014, Figure 1A. Furthermore, Figure 1 shows that the prevalence of PCR-determined *P. falciparum* positivity on day 3 by study year in patients aged < 5 years (Figure 1B) was slightly higher but not statistically significantly different from that of the all ages (Figure 1A) and of those aged > 5 years (Figure 1C).

### Prevalence of *Pfmdr1* N86Y and *Pfcrt* K76T on day 3.

**Figure 2.**
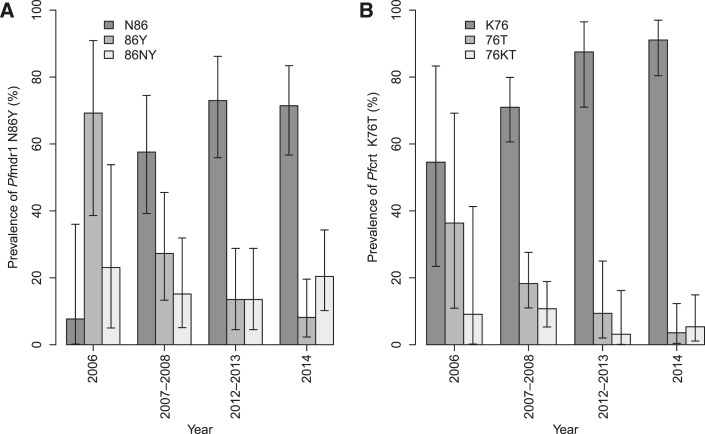
Distribution of *Pf*mdr1 N86Y (**A**) and *Pf*crt K76T (**B**) on day 3 across years of study.

A total of 131/256 (51.2%) and 192/256 (75.0%) samples were PCR positive on day 3 for *Pfmdr1* N86Y and *Pfcrt* K76T, respectively. The prevalence of *Pfmdr1* N86Y and *Pfcrt* K76T during the study period is presented in Figure 2. The prevalence of *Pfmdr1* N86 increased significantly from 7.7% (1/13) in 2006 to 57.6% (19/33) in 2007–2008, 72.9% (27/37) in 2012–2013, and 71.4% (35/49) in 2014 (odds ratio [OR] = 1.2, 95% CI: 1.09–1.33, *P* < 0.001), whereas that of *Pfcrt* K76 increased significantly from 54.5% (6/11) in 2006 to 70.9% (66/93) in 2007–2008, 87.5% (28/32) in 2012–2013, and 91.1% (51/56) in 2014 (OR = 1.3, 95% CI: 1.11–1.44, *P* < 0.001).

### Changes of *Pfmdr1* N86Y and *Pfcrt* K76T between pretreatment and on day 3 across years of study.

There was no significant difference in the change of *Pfmdr1* N86 to 86Y or N86Y, *Pfmdr1* 86Y to N86 or N86Y, and from *Pfmdr1* N86Y to N86 or 86Y (McNemar χ2 = 0.39, *P* = 0.5316) between pretreatment and day 3 across years of study. Similarly, the change of *Pfcrt* K76 to 76T or K76T, *Pfcrt* 76T to K76 or K76T, and from *Pfcrt* K76T to 76K or 76T between pretreatment and day 3 were not significantly different (McNemar χ2 = 0.25, *P* = 0.6171).

### Association between pretreatment characteristics and PCR-determined *P. falciparum* positivity on day 3.

Results for logistic regression of univariate and multivariate analyses for association between pretreatment characteristics and day 3 PCR-determined *P. falciparum* positivity are presented in Table 2. The years of study 2007–2008 and 2012–2013 had significantly higher odds of PCR-determined *P. falciparum* positivity on day 3 with the OR of 6-folds (*P* < 0.001) and 3-folds (*P* = 0.017), respectively, as compared with data from 2006. Other predictors for PCR-determined *P. falciparum* positivity were microscopy-determined asexual parasite density ≥ 100,000/µL (*P* < 0.001), hemoglobin < 10 g/dL (*P* = 0.025), age < 5 years (*P* < 0.001), and fever (axillary temperature ≥ 37.5°C) at inclusion (*P* = 0.043). Both *Pfmdr1* N86 (*P* = 0.71) and *Pfcrt* K76 (*P* = 0.72) were not significantly associated with PCR-determined *P. falciparum* positivity on day 3.

**Table 2:** Association between pretreatment characteristics and polymerase chain reaction–determined *Plasmodium falciparum* positivity on day 3

Characteristic	Univariate analysis odds ratio (95% CI)	*P*-value	Multivariate analysis adjusted odds ratio (95% CI)	*P*-value
Year 2006	1.0 (–)	–	1.0 (–)	–
2007–2008	7.4 (3.7–14.8)	< 0.001	6.3 (3.3–13.1)	< 0.001
2012–2013	1.4 (0.7–2.9)	0.31	2.9 (1.2–7.0)	0.017
2014	0.9 (0.5–2.0)	0.96	1.3 (0.6–2.8)	0.50
Microscopy-determined parasite density ≥ 100,000/µL	2.6 (1.6–4.0)	< 0.001	2.3 (1.4–3.8)	< 0.001
Hemoglobin level < 10 g/dL	2.4 (1.7–3.4)	< 0.001	1.5 (1.1–2.3)	0.025
Age < 5 years	3.8 (2.6–5.4)	< 0.001	3.1 (2.1–4.6)	< 0.001
Fever (≥ 37.5°C)	1.7 (1.1–2.5)	0.008	1.5 (1.0–2.3)	0.043
*Pfmdr1* N86	1.2 (0.8–1.9)	0.403	1.1 (0.7–1.8)	0.71
*Pfcrt* K76	0.5 (0.3–1.2)	0.113	0.9 (0.4–1.9)	0.72

### Association between PCR-determined *P. falciparum* positivity on day 3 and recurrent infection.

A total of 534 patients screened for PCR-determined *P. falciparum* positivity on day 3 were included in this analysis. Twenty-seven of 534 (5.1%) were either lost to follow-up or withdrew their consent between days 3 and 28. Of the remaining 507 patients, 44 (8.7%) had microscopy-determined recurrent infection, of whom seven (1.4%) had PCR-adjusted recrudescence. Of the 507 patients, 227 (44.8%) had PCR-determined *P. falciparum* positivity on day 3, of which 24 (10.6%) had microscopy-determined recurrent infection (χ2 = 1.86, *P* = 0.173) and four (0.018%) had PCR-adjusted recrudescence (Fisher’s exact test, *P* = 0.706).

## DISCUSSION

This study conducted in Bagamoyo district, Tanzania, where presently no data exist on presence of *PfK13* polymorphisms associated with artemisinin resistance, showed that more than one-third of the patients tested positive for *P. falciparum* by PCR on day 3, whereas only two patients had day 3 microscopy-determined asexual parasites. Importantly, the prevalence of day 3 PCR-determined *P. falciparum* positivity across study years in patients aged < 5 years was slightly higher but not statistically significantly different from that of all ages and of those aged > 5 years. Parasite clearance following treatment is determined by many factors including the mode of action of the used antimalarial drug and pretreatment characteristics of both the parasite and host.^17,18,21,35,36^ The day 3 PCR-determined *P. falciparum* positivity observed in this study might have also been present during the chloroquine (CQ) and sulfadoxine–pyrimethamine era because of their modes of actions, resulting in a relatively slow parasite clearance rate as compared with artemisinin.^35,36^ In the present study, the day 3 PCR-determined *P. falciparum* positivity might represent a remaining fraction of the parasite biomass that was then probably cleared by lumefantrine, the long-acting partner drug.^37^ Our observations are substantiated by the presence of a significant proportion of patients in the 2006 trial with PCR-determined *P. falciparum* positivity on day 3, despite being treated for uncomplicated *P. falciparum* malaria 6 months before the implementation of artemether–lumefantrine policy in Bagamoyo district. However, a study in Kenya found an association of the presence of PCR-determined *P. falciparum* parasites on day 3 with parasite tolerance against ACT.^16^ Nevertheless, differences in parasite detection methods between the studies with the use of quantitative-PCR (qPCR) in the Kenyan study might explain the differences observed between these two studies.^16^ Further surveillance of PCR-determined *P. falciparum* positivity on day 3 to provide a better understanding of this phenomenon is, therefore, warranted. On the other hand, the proportion of PCR-determined *P. falciparum* positivity on day 3 increased between 2006 and 2007–2008, and, thereafter, declined toward 2014. Differences in methods used may have resulted in different findings between the individual trials in this report. However, with the exception of a different DNA extraction method used for the 2006 trial, the amount of blood collected, type and storage of a blotted filter paper, and amplification methods were the same for all the included trials. Therefore, it is not well understood why the 2007–2008 trial had a high proportion of day 3 PCR-determined *P. falciparum* positivity as compared with other trials included in the analysis.

After data were pooled, higher initial microscopy-determined asexual parasitemia (≥ 100,000/µL of blood), hemoglobin < 10 g/dL, age < 5 years, body temperature ≥ 37.5°C, and the year of study were found to be associated with the presence of PCR-determined *P. falciparum* positivity on day 3. However, when the respective trials were analyzed separately, the same factors were significantly associated with PCR positivity on day 3 in some trials, but not in others. Contrary to our findings, a study in Kenya did not observe any association of pretreatment characteristics with qPCR-determined parasite positivity on day 3.^16^ However, previous studies have shown pretreatment characteristics of both the host and parasite, including existing immunity, pretreatment parasitemia, parasite strains, parasite developmental stage, and parasite drug susceptibility, to affect microscopy-determined parasite clearance time.^17,18,21,38,39^

*Pfmdr1* N86 and *Pfcrt* K76 proportions increased significantly across years, in line with previous reports.^40,41^ However, the presence of *Pfmdr1* N86 and *Pfcrt* K76 alleles in this study was not associated with a statistically significant increase in the presence of PCR-determined *P. falciparum* positivity on day 3. Furthermore, the changes between days 0 and 3 of *Pfmdr1* N86Y and *Pfcrt* K76T outcome were not significantly different across years. The absence of significant change of these alleles, particularly of *Pfmdr1* N86 and *Pfcrt* K76, between days 0 and 3 probably indicates that more time than 3 days is required for the parasite to be exposed to the drug for such selection to occur. Conversely, the relatively high proportions of *Pfmdr1* 86Y and *Pfcrt* 76T in 2006 probably reflect previous exposure of the parasite population to amodiaquine and CQ.^42–44^

The PCR-determined *P. falciparum* positivity on day 3 was neither associated with the microscopy-determined recurrent infections nor the PCR-adjusted recrudescence during follow-up after artemether–lumefantrine treatment. Furthermore, a large majority (83.3%) of the recurrent infections were due to reinfection based on PCR-adjustment. Similar findings have been reported in Uganda.^45^ A study in Kenya, however, reported the association of PCR-determined *P. falciparum* positivity with PCR-adjusted recrudescence.^16^ Nonetheless, differences in the methods used to determine parasites on day 3 and differentiate recrudescence from reinfection could probably account for the differences in the findings between the two studies.^16,29^ Unlike our study that determined day 3 *P. falciparum* positivity using conventional PCR, the study in Kenya used qPCR to determine both *P. falciparum* positivity and density.^16^ The qPCR is highly sensitive compared with the conventional PCR. The high qPCR-determined median parasite density in the Kenyan study, therefore, probably led to the observed association of day 3 parasitemia with PCR-adjusted recrudescence. Furthermore, in the Kenyan study, the standard WHO criteria to differentiate PCR-adjusted recrudescence from the new infection was modified, whereby samples obtained 1 day before and on the day of recurrent infection were compared with those of days 1 and 2 in addition to the baseline samples.^16,29^

The strength of this study is that it included four studies conducted over different years, before and up to 8 years after implementation of artemether–lumefantrine as first-line treatment of uncomplicated malaria in Bagamoyo district. However, study limitations include, for example, that no blood sampling was performed to assess whether the PCR-determined *P. falciparum* parasites were cleared during follow-up between days 4 and 7. Moreover, neither was any evaluation of in vitro responsiveness to artemether–lumefantrine carried out nor assessment of day 7 lumefantrine concentration levels.

## CONCLUSION

Day 3 PCR-determined *P. falciparum* positivity remained common in patients treated with artemether–lumefantrine before and after implementation of ACT as policy in Bagamoyo district, Tanzania. However, its presence was associated with pretreatment characteristics including high pretreatment asexual parasitemia (≥ 100,000/µL of blood), anemia (hemoglobin < 10g/dL), age < 5 years, documented fever at enrollment, and the year of study.
